# Leptin Stimulates Cellular Glycolysis Through a STAT3 Dependent Mechanism in Tilapia

**DOI:** 10.3389/fendo.2018.00465

**Published:** 2018-08-21

**Authors:** Jonathan D. Douros, David A. Baltzegar, Benjamin J. Reading, Andre P. Seale, Darren T. Lerner, E. Gordon Grau, Russell J. Borski

**Affiliations:** ^1^Department of Biological Sciences, North Carolina State University, Raleigh, NC, United States; ^2^Genomics Sciences Laboratory, North Carolina State University, Raleigh, NC, United States; ^3^Department of Applied Ecology, North Carolina State University, Raleigh, NC, United States; ^4^Hawaii Institute of Marine Biology, University of Hawaii, Kaneohe, HI, United States; ^5^Department of Human Nutrition, Food, and Animal Sciences, University of Hawaii at Mānoa, Honolulu, HI, United States; ^6^University of Hawaii Sea Grant College Program, Honolulu, HI, United States

**Keywords:** leptin, prolactin cell, hepatocytes, pituitary, RNAseq, fishes, phosphofructokinase-1

## Abstract

We assessed if leptin, a cytokine hormone known to enhance energy expenditure by promoting lipid and carbohydrate catabolism in response to physiologic stress, might directly regulate cellular glycolysis. A transcriptomic analysis of prolactin cells in the tilapia (*Oreochromis mossambicus*) pituitary rostral pars distalis (RPD) revealed that recombinant leptin (rtLep) differentially regulates 1,995 genes, *in vitro*. Machine learning algorithms and clustering analyses show leptin influences numerous cellular gene networks including metabolism; protein processing, transport, and metabolism; cell cycle and the hypoxia response. Leptin stimulates transcript abundance of the glycolytic enzyme glyceraldehyde-3-phosphate dehydrogenase (*gapdh*) in a covariate manner to the hypoxic stress gene network. Orthogonal tests confirm that rtLepA dose-dependently increases *gapdh* gene expression in the RPD along with transcript abundance of 6-phosphofructo-1-kinase (*pfk1*), the rate limiting glycolytic enzyme. Functional testing demonstrated that leptin stimulates PFK activity and glycolytic output, while Stattic (a STAT3 blocker) was sufficient to suppress these responses, indicating leptin stimulates glycolysis through a STAT3-dependent mechanism. Leptin also stimulated *pfk1* gene expression and lactate production in primary hepatocyte incubations in a similar manner to those shown for the pituitary RPD. This work characterizes a critical metabolic action of leptin to directly stimulate glycolysis across tissue types in a teleost model system, and suggest that leptin may promote energy expenditure, in part, by stimulating glycolysis. These data in a teleost fish, suggest that one of leptin's ancient, highly-conserved functions among vertebrates may be stimulation of glycolysis to facilitate the energetic needs associated with various stressors.

## Introduction

Leptin is a pleiotropic cytokine that regulates numerous physiological processes and whose dysfunction is implicated in pathologies including obesity, diabetes, and cancer (1, 2). Leptin has been classically described as an adipostat in mammals, whereby the hormone circulates in proportion to total body lipid content, while acting to promote satiety and energy expenditure through stimulation of metabolic rate and fatty acid oxidation (3, 4), and thereby, preventing excessive lipid accumulation. Impairment of leptin production or signaling leads to obesity and reduced metabolic rate (5). Additional evidence in both mammalian and teleost fishes suggest leptin may stimulate energy expenditure through carbohydrate catabolism as the hormone promotes hepatic glycogenolysis and peripheral glucose uptake (6–8), while functional leptin receptor (*lepr*) ablation in zebrafish drives β-cell hyperplasia, hyperglycemia, and an impairment of glucose control (9). It is clear that leptin serves to regulate both lipid and carbohydrate energy stores, however the physiologic relevance of this regulation remains poorly characterized. Hyperosmotic perturbation increases glucose mobilization (glycogenolysis), and metabolic rate (O_2_ consumption) in addition to stimulating leptin expression. Studies demonstrate that teleost leptin stimulates hepatic glycogenolysis and overall metabolic rate during stress (5, 8, 10, 11). Paradoxically, leptin is elevated during hypoxia in a wide range of vertebrates, when metabolic rate is suppressed; thus, the hormone may drive energy production and overall expenditure even when oxidative respiration is less viable, possibly by stimulating fermentation pathways (12–15). These findings point toward an emerging paradigm for leptin across vertebrate taxa as a comprehensive promoter of energy expenditure under both normoxic and hypoxic conditions where it mobilizes energy reserves, enhances substrate uptake in peripheral cells, and induces their catabolism. The full scope of molecular mechanisms by which leptin regulates energy expenditure remains unclear, however early studies suggest the hormone might stimulate glycolysis as measured by whole body conversion of glucose to water *in vivo*. While these studies suggest leptin stimulation of glycolysis is mediated, in part through central nervous system signals (7), the direct cellular effects of leptin on glycolytic output, the regulation of critical glycolytic genes, and the intracellular signaling mechanisms that mediate them are currently unknown.

Glycolysis, the enzymatic conversion of glucose to pyruvate, is the foundational biochemical pathway for ATP synthesis (16). This process is often regulated in concert with metabolic rate under conditions in which leptin also increases as a possible means to meet the energetic demands for maintaining homeostasis. Metabolic rate, glycolytic activity and leptin increase during periods of hyperglycemia associated with acute osmoregulatory stress as well as during tumorigenesis (8, 11, 17–21). On the other hand, metabolic suppression as seen with hypoxia and hibernation is also associated with enhanced glycolysis, as animals attempt to meet energy requirements through anaerobic pathways (12, 22). Evidence in a wide range of vertebrates also show that leptin increases with hypoxia (13–15). Metabolic regulation of glycolysis is mediated by an array of factors, including hormonal signals. For instance, sympathetic catecholamines act via cAMP to either stimulate (muscle) or inhibit (hepatic) glycolysis; while insulin and hypoxia-inducible factor 1 may work through AKT-signaling pathways or by inducing gene transcription of genes involved in glucose uptake and metabolism through signal transducers and activators of transcription (STATs), respectively, to stimulate glucose catabolism (23–28). Given the evidence indicating concordant regulation of leptin with enhanced glycolysis under various stressors, and the known role of STAT3 and STAT5 to mediate leptin signaling in glucose catabolizing tissues, we postulate that the hormone may act directly to stimulate glucose catabolism, thereby increasing energy production and expenditure, and that this basal action may provide the requisite energetic substrates to maintain cellular homeostasis.

We assessed herein, the direct effects of leptin on glycolysis in the tilapia (*Oreochromis mossambicus*) pituitary rostral pars distalis (RPD), which in most teleost fishes contains a nearly homogeneous population of prolactin producing cells that can be studied *in vitro* in their natural *in situ* aggregated form (29). This contrasts with the mammalian pituitary in which lactotrophs are interspersed with other pituitary cell types making them more difficult to isolate for study. Studies show that the tilapia prolactin cell, like that of other vertebrates, is also highly sensitive to stimulation by leptin, making this an excellent physiologic model for examining the discrete cellular actions of leptin (30, 31). We first characterized leptin-induced changes in global gene expression to ascertain potential cellular actions of leptin, using an advanced clustering analysis of the RPD transcriptome. We further tested the transcriptome findings by measuring leptin effects on gene expression and activity of glycolytic enzymes, total glycolytic activity, and STAT3 activation in a series of *in vitro* experiments using both pituitary RPD and hepatocyte incubations. The data illustrate leptin directly stimulates glycolysis by increasing the expression of glycolytic genes, the activity of glycolytic enzymes, and overall glycolytic output through a STAT3 dependent mechanism.

## Materials and methods

Adult male tilapia (~60–80 g) were housed in freshwater (FW) recirculating tank systems (salinity 0–0.5 ppt, hardness 74–84 mg/L, alkalinity 126–178 mg/L, pH 8.0) at 24–26°C with a photoperiod of 12:12 h of light and dark and fed daily (1–2% body weight/day). Fish were anesthetized in buffered tricaine methanesulfonate (MS-222) and decapitated prior to sampling of pituitary and liver. All animal protocols were approved by the NCSU Institutional Animal Care and Use Committee.

### Pituitary incubations

Procedures for pituitary RPD isolation and incubations have been described previously (30, 32). The pituitary was removed and the RPD was dissected and placed individually in wells of a 96-well plate containing Modified Kreb's bicarbonate Ringer (320 mOsmolal) solution with addition of glucose (5 mM), L-glutamine (2.05 mM), and Eagles MEM (Gibco, Grand Island, NY) whereby pH of the media was adjusted to 7.4. Tissues were incubated at 24°C in a humidified environment containing 95%O_2_/5%CO_2_ at 60 revolutions per minute. Following a 2-h preincubation, the media was replaced with fresh control or experimental media containing recombinant tilapia Leptin A (rtLepA, dosages indicated in figures and legends), the dominant leptin paralog in teleost fishes (30), and/or pharmacological agents at concentrations and time periods provided in the figures (33). Media was removed and frozen at −20°C for subsequent lactate measurement. Tissues were removed and placed in either RNALater at 4°C for assessment of gene expression or in phosphofructokinase (PFK) assay buffer (Sigma), snap-frozen in liquid nitrogen then placed at −20°C for later Western blot analyses and PFK activity determinations.

### Hepatocyte incubations

In order to establish the effects of leptin on another tissue type, we examined the hormone's regulation of hepatic lactate secretion, phosphofructokinase 1 (*pfk1*) gene expression and glyceraldehyde 6-phosphate dehydrogenase (*gapdh*) gene expression using *in vitro* hepatocyte incubations (30, 34). This entailed lethal anesthesia (MS-222) followed by removal of the liver, which was finely excised with a razor blade in a calcium-free Hank's buffered salt solution (HBSS) containing 0.3 mg/mL type IV collagenase (Sigma-Aldrich, St. Louis, MO). Tissues were then incubated in the HBSS-collagenase solution for 30–45 min at room temperature. The digested liver tissue was gently pushed through a 260 μm mesh filter to remove structural tissue, and then allowed to drip through a second 60 μm mesh. Filtered hepatocytes were collected in an ice-cold beaker then washed in HBSS containing 3 mM CaCl_2_ and 1X MEM solution, with essential and nonessential amino acids (Gibco), and allowed to recover on ice for 1 h. After centrifugation cells were resuspended in RPMI 1640 growth media containing L-glutamine (Gibco; 1% streptomycin/penicillin mixture added), at 315 mOsm. The hepatocytes were then plated on 24-well Falcon Primaria plates at a density of 3.5 × 10^5^ cells/mL. Cell viability was determined to be >90% using a Trypan Blue exclusion test. After plating, cells were allowed to acclimate for 4 h at 24°C in a humidified air atmosphere. After this preincubation period, the growth media were removed and replaced by experimental media (0, 1, 10, 100 nM rtLepA or control, *n* = 6/treatment, 4 groups) (30). Experimental incubations were carried out for 6-h at 24°C under ambient air conditions. At termination, media lactate was measured, and cells were collected in Tri-reagent and stored at 4°C until RNA isolation for gene expression analysis of *pfk1* and *gapdh*.

### Lactate and PFK activity

Media lactate secretion was assessed as a proxy for total glycolytic activity (35). Lactate released in media was quantified using a commercial colorimetric assay that measures NADH derived from lactate oxidation (Abcam, Cambridge, MA). PFK enzymatic activity was measured by a commercial colorimetric assay that directly quantifies ADP formation derived by conversion of PFK substrate, fructose-6-phosphate, to fructose-1,6-diphosphate (Sigma-Aldrich). Lactate secretion and PFK activity were normalized to tissue protein concentration as measured by bicinchoninic acid assay (BCA, Pierce^TM^, Grand Island, NY).

### Western blot assays

Cellular STAT3 proteins (phosphorylated and nonphosphorylated) were detected by Western blot and quantified using a LI-COR Odyssey infrared imaging system as described previously for tilapia (30, 33). Frozen tissues were sonicated on ice for 3 s in reducing sample buffer [NuPAGE, Invitrogen, San Diego, CA; final concentration in the loaded samples in mmol/l: 141 Tris base, 106 Tris HCl, 73 LDS, 0.5 EDTA, 50 1,4-dithiothretiol and 8% glycerol (v/v), 0.019% Serva blue G250 (w/v)], and total protein was assessed by BCA (Thermo Scientific). Samples (50 ug total protein) were loaded onto a 4–12% bis-tris gels (NuPAGE), and MES/SDS-buffer (in mmol/l: 50 2-(*N*-morpholino)-ethanesulfonic acid, 50 Tris, 3.5 SDS, 1 Na2-EDTA; with addition of NuPAGE antioxidant) at 200 V (Xcell II SureLock; Invitrogen). Proteins from gel were then transferred onto nitro-cellulose membranes (0.45 μm; Invitrogen) by submerged-blotting for 1 h at 30 V [XCell II; Invitrogen; (transfer buffer mmol/l: 25 Tris, 192 glycin and 20% methanol)]. Membranes were blocked in TBS-T with LI-COR (Lincoln, NE) blocking buffer (1:1) and washed in TBS-T. Phosphorylated (active) STAT3 was detected by a polyclonal anti-STAT3 p-Y705 (Abcam; AB76315) incubated overnight at 4°C with gentle shaking using 1:5000 dilutions. Total STAT3 protein was similarly detected using anti-STAT3 (Abcam; AB50761) at a 1:5000 dilution. Following washing in TBS-T, membranes were incubated at room temperature for 1 h with goat anti-rabbit secondary antibody conjugated to Alexa IRDye 680 (LI-COR). Protein detection and quantification was determined by the LICOR Odyssey. Background signal of blot was subtracted from that of phosphorylated STAT3 and total STAT3. Active (phosphorylated) STAT3 was then normalized to total STAT3 content, as appropriate for quantifying post-translationally modified signaling proteins.

### Single gene expression analysis by qPCR

Hepatic and pituitary RNA was isolated using TRI reagent (Molecular Research Center, Cincinnati, OH), coupled with on-column affinity purification and DNAse treatment (Direct-zol minipreps, Zymo Research Corporation, Irvine, CA) as described previously (8, 30). For gene expression studies using qRT-PCR, total RNA (0.8–1 μg) was reverse transcribed to cDNA using random priming hexamers (High Capacity cDNA Synthesis kit, Life Technologies). Gene expression of *pfk1* (XM_019349871, Forward primer: 5′-ACAGATCGAGCCCCTTACCT-3′, Reverse primer: 5′-ACGGTGGCAGGTATAACCAC-3′), *gapdh* (XM_003443681, Forward primer: 5′-ATCTCTGTCTTCCAGTGTATGAAGC-3′, Reverse primer: 5′-CTCATTAACTCCCATGACAAACATT-3′), *prl1* (M27010, Forward primer: 5′-TCT GAC AAA CTG CAC TCG CT-3′, Reverse primer: 5′-TCC TTG TCA ATG GGC GTC TG-3′), and *prl2* (M27011, Forward primer: 5′-GTT GTG TGT GGT GGC AAT GT-3′, Reverse primer: 5′-TTG TCA GTG GGC GTC TGT AG-3′) were quantified via SYBR green qPCR on an ABI 7900HT sequence detection platform using duplicate runs for all samples, standards, and negative controls: 1 cycle-95°C for 10 min; 40 cycles-95°C for 30 s, 60°C for 60 s, and 72°C for a 60 s extension. Gene expression was then normalized to 18S ribosomal RNA as described previously (30).

### RNA-seq and bioinformatic analyses of the pituitary transcriptome

For RNA-seq studies assessing leptin modulation of the pituitary RPD transcriptome, tissues were placed in wells of a Falcon 6-well plate in 3 mL of either hormone-free (control) or leptin-containing medium and incubated for 6 h (*n* = 30/treatment; 10 RPD/well, 3 wells/treatment). Tissues from each well were pooled in 2 mL of RNAlater at 4°C until RNA isolation and Illumina cDNA library preparation.

Pituitary mRNA (10 μg) was submitted to the North Carolina State University Genomic Sciences Laboratory (Raleigh, NC) for Illumina cDNA library construction. The cDNA was synthesized using the SuperScript Double-Stranded cDNA Synthesis Kit (Invitrogen) and tagged with a 5′, four-nucleotide barcode. Illumina cDNA libraries (*n* = 3) were prepared for each treatment (control or leptin). Sequencing was performed in triplicate at the Hawaii Institute of Marine Biology Genetics core facility on an Illumina MiSeq platform, using 150 bp, paired-end reads.

The FASTQ sequencing output files from each group were trimmed for barcode removal and standard quality control (Phred score > 36; FastQC) and, then aligned to the *Oreochomis niloticus* genome (Ensembl v. 1.0.71) using a local, short read aligner (Bowtie 2-2.1) (36, 37). The fragments per kilobase of exon per million fragments mapped (FPKM, *i.e*. copy number) were calculated for all annotated genes using Cufflinks-2.2.1. Statistical comparisons between control and leptin treatments were made using Cuffdiff-2.2.1 to determine significant differentially expressed genes (38–41).

Reduction of data dimensionality was conducted in order to empirically determine which differentially expressed genes (DEGs) are required to build a predictable model of leptin effects on the pituitary transcriptome (i.e., “highly predictive” DEG). This was accomplished by inputting FPKMs of each DEG into a multilayer perceptron (WEKA software) (42) and generating artificial neural networks (i.e., model for leptin signaling). Modulated Modularity Clustering (MMC) was used to group highly significant DEGs into small “modules” (i.e., subsets of covariable, functionally interactive genes) (43). RNA-seq was assessed statistically within Cuffdiff-2.2.1 followed by Benjamini-Hochberg correction (39, 41). The residuals of each highly significant DEG are input into the MMC program. The Gene Ontology (GO) term enrichment (i.e., function) of each covariable module was assessed using the DAVID Bioinformatics pathway suite (44). Subsequent visualizations of each module were constructed using Cytoscape (45).

### Statistics

The time course experiment was analyzed using two-way ANOVA (treatment x time) followed by Tukey's HSD *post-hoc* test (GraphPad Prism). All other statistics were assessed using one-way ANOVA followed by Tukey's HSD *post-hoc* test. Linear regression analysis was performed to assess the relationship between lactate secretion and PFK activity and *pfk* gene expression as well as *gapdh* gene expression; a significant relationship denotes a slope that differs from zero with *p* < 0.05 (GraphPad Prism).

## Results

### Effects of leptin on the pituitary transcriptome

**Figure 1:**
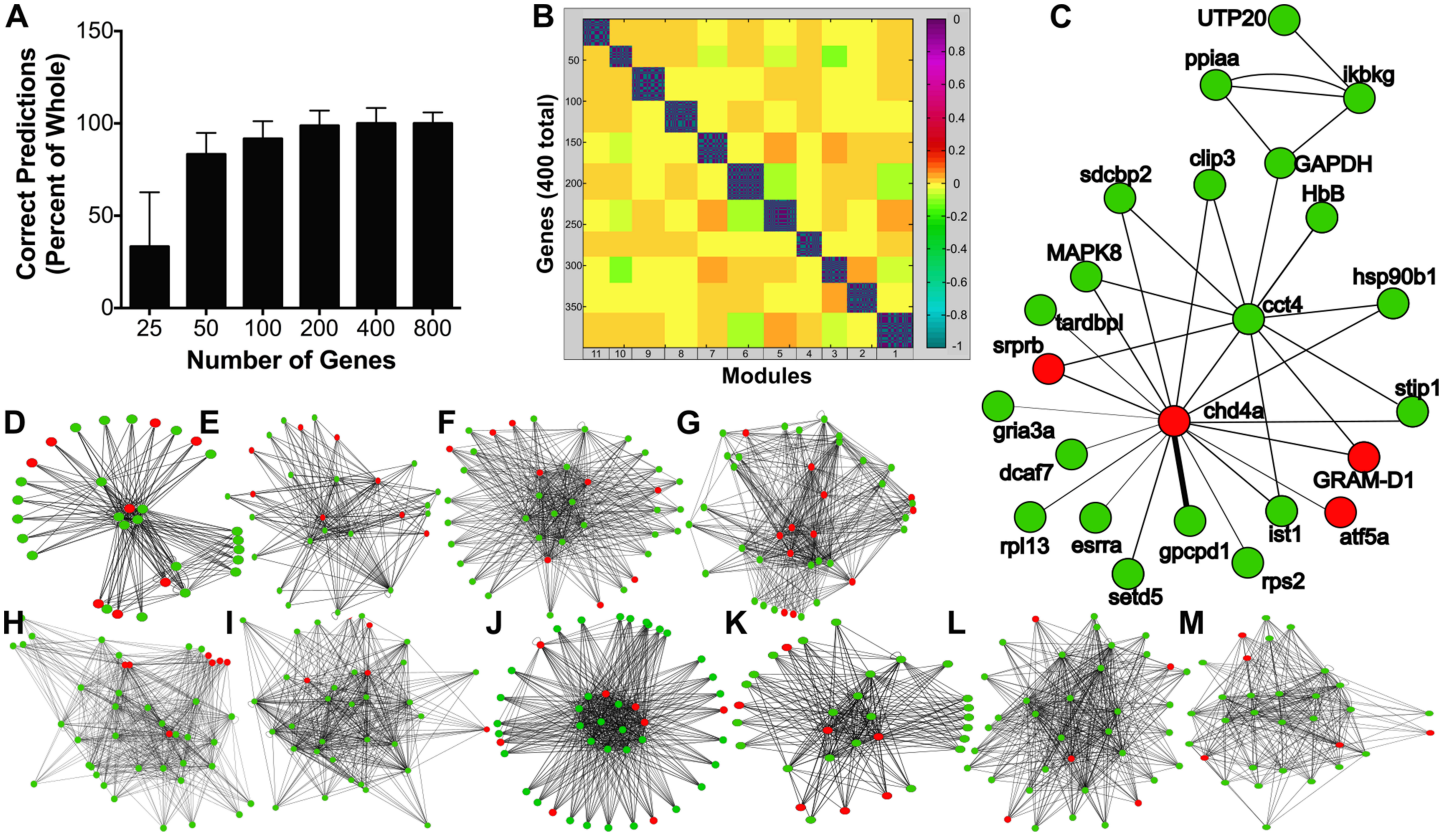
**(A)** Reduction of data dimensionality reported as the accuracy (percentage) with which the multilayer perceptron (machine learning program) is able to learn and recognize leptin modulation of the pituitary RPD transcriptome given as a function of the number of gene inputs (FPKM of DEGs given in triplicate). **(B)** MMC heat map of 400 correlated genes displaying 11 modules. **(C–M)** Cytoscape diagrams represent the genes within each module and their interactions (Module 1–6 correspond to D–I, 8–11, to J–M). More detailed cytoscape diagrams are presented in Supplemental Figure 1. Module 7 **(C)** represents “Regulation of Hypoxia” GO term enrichment. Green circles represent upregulated and red circles downregulated genes. The genes involved and the GO term enrichment of each module can be found in Supplemental Table 1 and Table 1, respectively.

The effect of homologous recombinant leptin A (LepA, 100 nM) on the global gene expression profiles of the tilapia pituitary RPD transcriptome was examined using an RNA-seq approach (accession number GSE117682). Illumina sequencing of the RPD returned ~17 million paired-end reads of 150 bp per sample which aligned to the *O. niloticus* reference genome with >93% accuracy. The rtLepA treatment significantly regulates 1,995 genes (1,284 stimulated; 711 suppressed) compared with controls (*p* < 0.05; Differentially Expressed Genes [DEGs], Supplemental Table 1). One glycolytic gene of particular interest for further study, *gapdh*, was stimulated 2.25-fold. To identify functionally interactive gene networks regulated by leptin, a reduction of data dimensionality (RDD) analyses was performed on the most highly significant DEGs or those with the lowest *p*-values. The RDD demonstrates that at least 400 highly significant DEG inputs are required to build an artificial neural network model of leptin signaling with optimal predictive accuracy (Figure 1A; Supplemental Table 2). Modulated modularity clustering (MMC) groups the 400 highly significant DEGs into 11 modules predicted to be functionally interactive (Figures 1C–M). The GO term enrichment of the 11 MMC modules is reflective of pathways regulated by leptin and includes metabolism, translational processes (protein processing, transport, and metabolism), cell division, hypoxia response, and others (GO terms, Table 1; MMC modules, Supplemental Tables 3, 4, Supplemental Figure 1).

**Table 1:** Gene Ontology enrichment of modulated modularity clusters.

Process/Module^*^	**Function (GO term number)**
**METABOLISM**	
11	Pyruvate metabolism (0006090)
5	Phosphorus/phosphate metabolism (0006793/0006796); negative regulation of macromolecule metabolism (0010605)
**TRANSLATION**	
1	Regulation of translational initiation (0006446)
1,4,5,6,8,11	Translation (0006412)
1,5,6,8,11	Translational initiation (0006413)
8	tRNA amino acylation for protein translation (0006418)
**PROTEIN PROCESSING**	
2,4	Protein localization (0008104)
5	Regulation of protein complex disassembly (0043244); protein amino acid phosphorylation (0006468)
6	Protein folding (0006457)
**PROTEIN TRANSPORT**	
2	Intracellular transport (0046907); vesicle-mediated transport (0016192); endosome transport (0016197)
4	Protein transport (0015031); establishment of protein localization (0045184)
**PROTEIN METABOLISM**	
5	Regulation of proteolysis (0030162); negative regulation of cellular protein metabolism (0032269/0051248); phosphorylation (0016310)
8	Amino acid activation (0043038); tRNA amino acylation (0043039)
10	Cytokine-mediated signaling pathway (0019221)
**CELL CYCLE**	
4	Cell cycle (0007049); cell division (0051301)
**Hypoxia response**	
7	Response to oxygen levels/hypoxia (0070482/0001666)

* *Module 3 is not enriched for any Gene Ontology terms*.

### Effect of leptin administration on glycolysis

**Figure 2:**
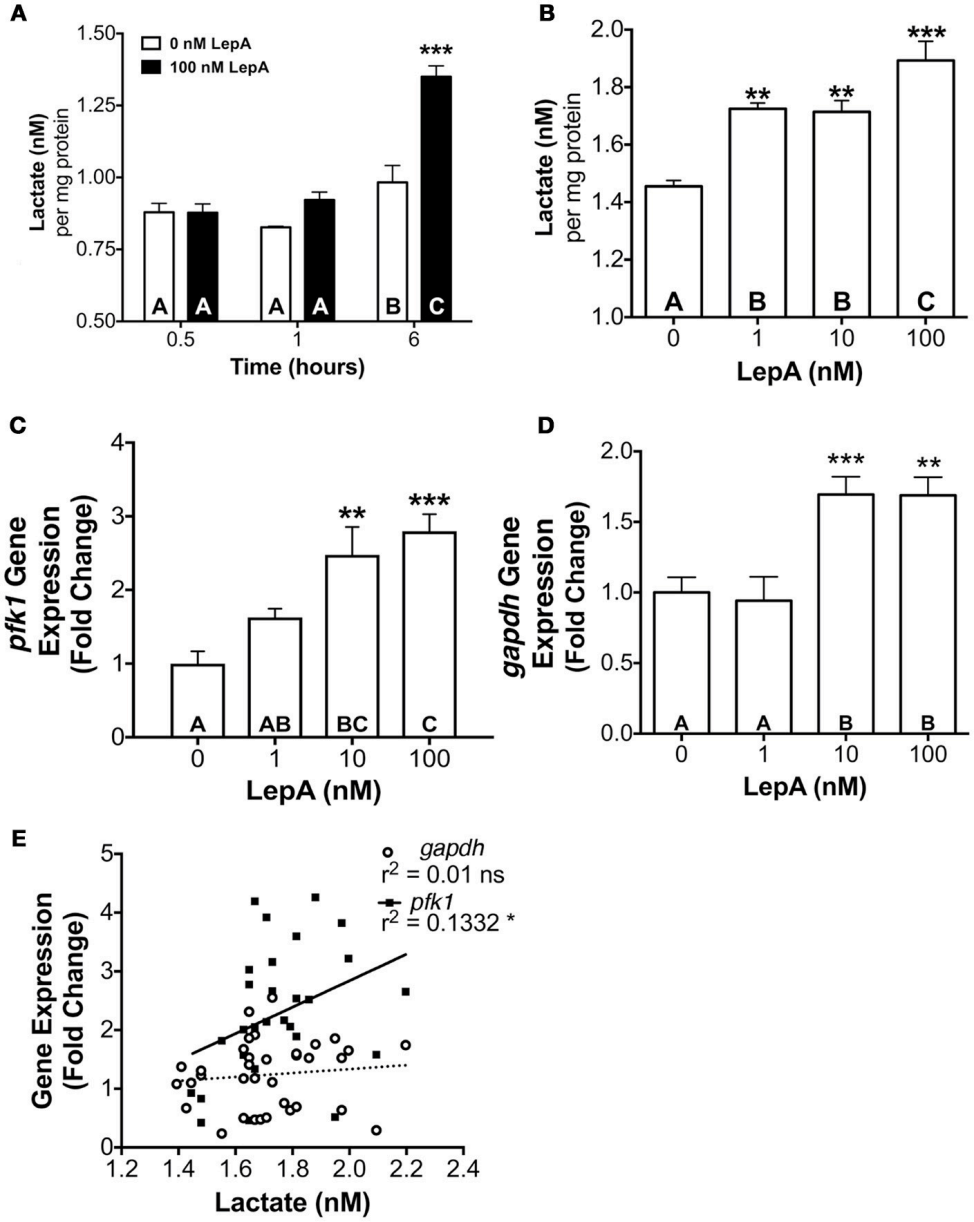
**(A)** Effect of leptin (100 nM) on lactate secretion from pituitary RPDs following 0.5, 1.0, and 6.0 h incubation (*n* = 5/group; 0 nM and 100 nM rtLepA). *In vitro* pituitary **(B)** lactate secretion, **(C)** PFK1 mRNA, **(D)** GAPDH mRNA abundance, and **(E)** correlation between lactate secretion and PFK and GAPDH mRNA abundance in response to rtLepA treatment (0, 1, 10, 100 nM) after 6 h incubation (*n* = 10/group). Asterisks denote significance differences relative to control (mean ± SEM; ^*^p < 0.05, ^**^p < 0.01, ^***^p < 0.001). Groups with different letters denote significant differences from each other.

Based on regulation of genes associated with hypoxia and the stimulatory effects of leptin on glycolytic enzyme gene expression observed from the transcriptomic analyses, we hypothesized leptin might increase glycolysis in the pituitary. A time course and dose response study was conducted to evaluate leptin regulation of glycolytic activity in RPD. Lactate secretion, a proxy for glycolytic activity (35), was not affected at 0.5 and 1.0 h but significantly increased following 6 h of exposure to 100 nM rtLepA (*p* < 0.0001; Figure 2A). Lactate secretion increased by 18–30% with rtLepA at concentrations of 1, 10, and 100 nM following 6 h incubations (*p* < 0.005; Figure 2B).

The changes observed in glycolysis were accompanied by elevations in both *gapdh* and *pfk1* mRNA expression. Leptin dose-dependently increased *pfk1* and *gapdh* mRNA by >2.5- and 1.7-fold, respectively (*p* < 0.01; Figures 2C,D). Lactate secretion correlated significantly to *pfk1*, but not *gapdh* gene expression (r^2^ = 0.1332; *p* < 0.05; Figure 2E).

**Figure 3:**
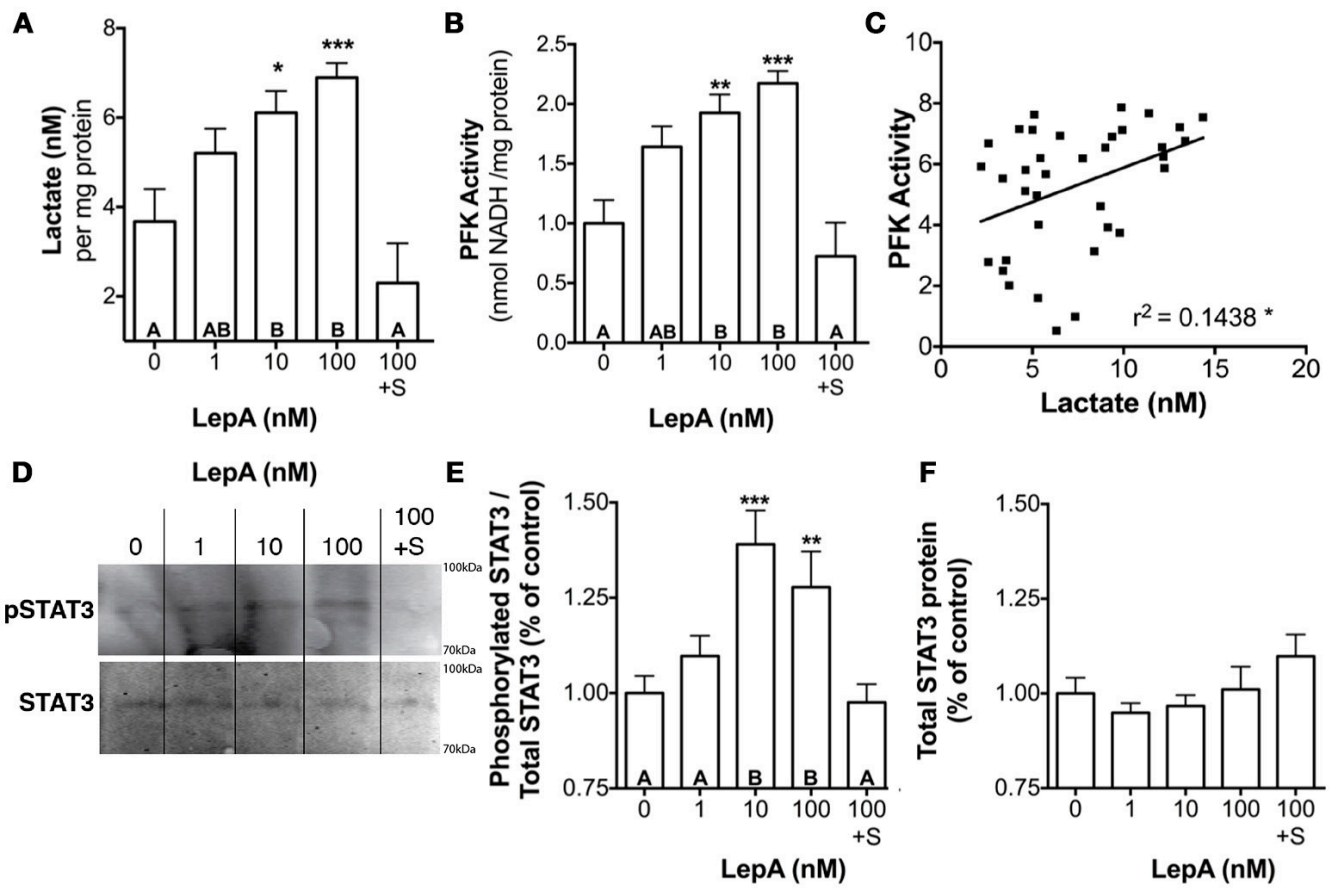
Pituitary RPD **(A)** lactate secretion, **(B)** PFK activity, **(E)** phosphorylated STAT3 (STAT3 activation), and **(F)** total STAT3 in response to rtLepA treatment (0, 1, 10, 100 nM), rtLepA (100 nM) + Stattic (S; 1 mM) (n = 6/treatment, 6 h incubation). **(C)** Correlation between lactate secretion and PFK activity in response to rtLepA treatment after 6 h. **(D)** a representative Western blot of phosphorylated STAT3 (relative to total STAT3) and total STAT3 in response to rtLepA as measured by infrared imaging. Asterisks denote significance differences relative to control (mean ± SEM; ^*^p < 0.05, ^**^p < 0.01, ^***^p < 0.001). Groups with different letters denote significant differences from each other.

The actions of leptin occur through binding a cytokine type 1 leptin receptor (LepR) that, in turn, leads to the activation of Janus Kinase (JAK)-STAT signaling pathways, with JAK2-STAT3 being an important transducer of leptin actions on appetite and energy expenditure (46, 47). Therefore, we evaluated if STAT3 might be an important mediator of the glycolytic actions of leptin. Studies assessed the effect of leptin on activation of STAT3 and of a STAT inhibitor on glycolytic output and PFK activity. Leptin at 10 and 100 nM concentrations stimulates lactate secretion by 66–87% (*p* < 0.05; Figure 3A) and PFK enzyme activity by 92–117% (*p* < 0.001; Figure 3B), respectively relative to controls. Coincubation of RPDs with rtLepA and the STAT3 activation and dimerization inhibitor Stattic (48) significantly suppresses both lactate secretion and PFK activity by > 75% compared to rtLepA treatment alone (*p* < 0.0001; Figures 3A,B). Pituitary lactate secretion significantly correlates with PFK activity (Figure 3C, r^2^ = 0.1438). LepA treatments significantly enhanced STAT3 activity, as measured by phosphorylation, by ~25-40% (Figures 3D,E). The total amount of STAT3 was not significantly altered by rtLepA (Figures 3D,F).

### Effect of leptin on hepatic glycolysis

**Figure 4:**
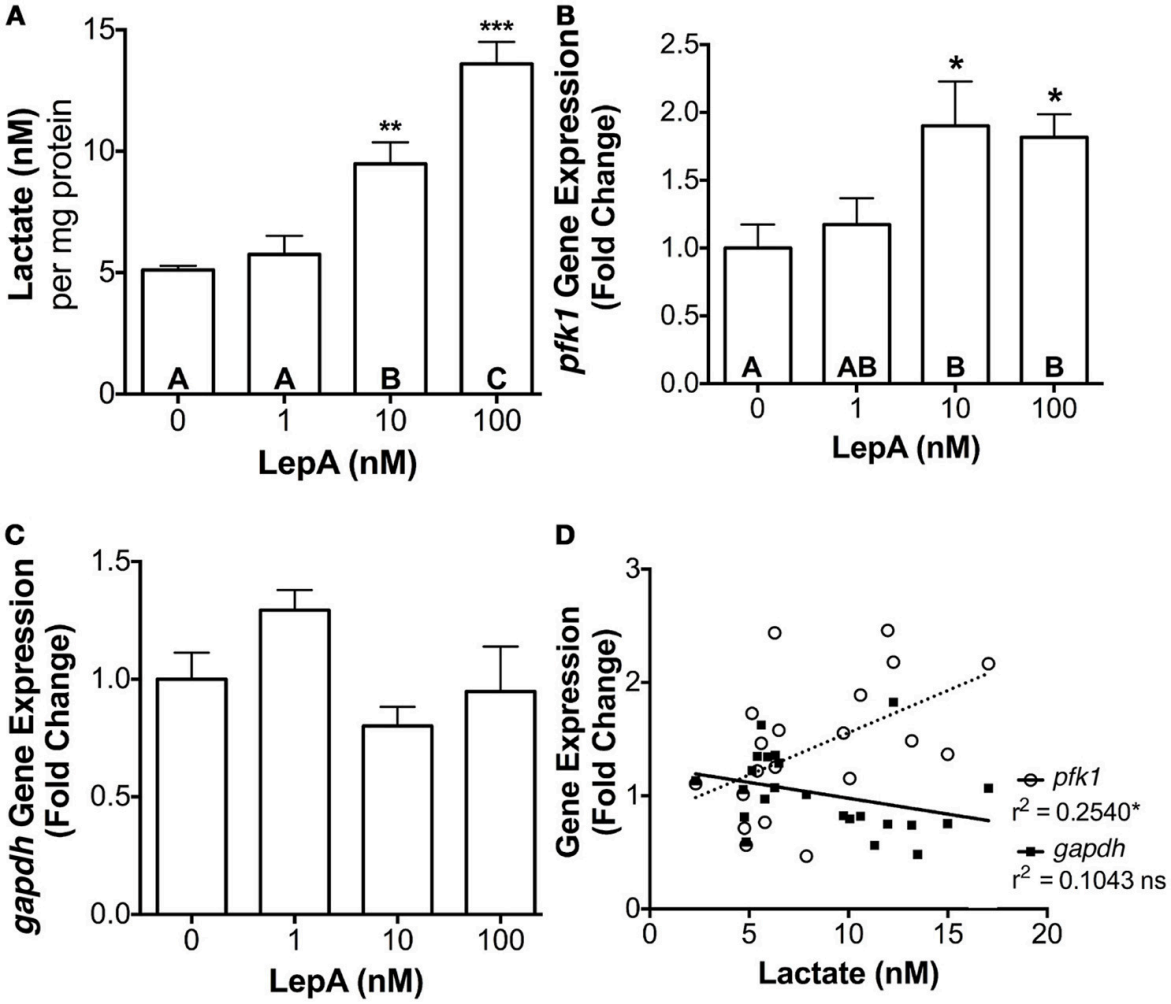
Hepatocyte **(A)** lactate secretion, **(B)** *pfk1* mRNA, and **(C)** *gapdh* mRNA abundance in response to rtLepA (0, 1, 10, 100 nM) treatment. **(D)** Correlation between lactate secretion and *pfk1* and *gapdh* mRNA abundance. Hepatocytes were plated at a density of 3 × 10^5^ cells per well (*n* = 6/treatment) and incubated for 6 h. The mRNA abundance *pfk1* and *gapdh* was normalized to 18s RNA and values reflect fold change relative to control (0 rtLepA). Asterisks denote significance differences relative to control (mean ± SEM; ^*^p < 0.05, ^**^p < 0.01, ^***^p < .001). Groups with different letters denote significant differences from each other.

To determine if the leptin's actions on the glycolytic pathway might be broadly applicable to other cell types, its effects on lactate secretion and *pfk1* and *gapdh* gene expression was assessed in primary hepatocytes. Leptin was effective in stimulating *pfk1* gene expression by > 80% (*p* < 0.05) and lactate secretion by >85% (*p* < 0.005; Figures 4A,B), but was ineffective in altering *gapdh* gene expression (Figure 4C). As observed with prolactin cells, hepatic lactate secretion correlated significantly with *pfk1* expression (r^2^ = 0.2540; *p* < 0.05; Figure 4D).

## Discussion

The potential action for leptin in regulating various cellular processes, including glycolysis, was identified utilizing an advanced neural network analyses of transcriptomic data. *In vitro* leptin administration differentially regulates 1,995 genes (Supplemental Table 1) in the pituitary RPD, which contains greater than a 95% pure population of prolactin cells (49). Of these 1,995 DEGs, 1,284 (64.3%) are up-regulated, while only 711 (35.6%) are down-regulated, suggesting leptin exerts largely stimulatory actions on pituitary gene expression consistent with that observed for other stress signals (50, 51). We further assessed potential functional effects of leptin by machine-learning algorithms (Multilayer Perceptron; MLP) that identified 400 highly significant DEGs predicted to be central to leptin action (Supplemental Table 2; Figure 1A)(42, 52, 53). These 400 highly significant DEGs cluster into 11 covariate modules predicted to be functionally interactive using MMC (Figure 1B) (43, 54). These modules are involved in a variety of broad cellular functions, including: metabolism, protein processing, cell cycle, cell proliferation, hypoxia adaptation, among others (Table 1). The hypoxic adaptation module (Module 7: Figure 1C) contains the glycolytic enzyme GAPDH along with many known hypoxic and hyperosmotic-sensitive genes including: chaperonin containing TCP1 (*cct*), chromodomain-helicase-DNA-binding protein 4 (*chdb-4*), glycerophosphocholine phosphodiesterase (*gpcpd1*), increased sodium tolerance-1 (*ist1*), heat shock protein beta 1 (*hsp90b*), inhibitor of NF Kappa B kinase (*ikbkg*), peptidylprolyl isomerase A (*ppiaa*), and ribosome assembly subunit (*utp20*) (Supplemental Table 3). The genes *cct, hsp90*, and *ppiaa* in particular, are known to promote protein folding thereby acting to protect protein tertiary structure under extreme cellular conditions and suggesting a mechanism for leptin stimulated hypoxic and osmotic adaptation (55–57). Additionally, a notable cluster-to-cluster interaction is observed between module 7 (hypoxia adaptation) and 10 (cytokine signaling) (i.e., high-coverability between different gene clusters as determined by Pearson correlation coefficient > 0.95, data note shown). This indicates that the gene transcription programs initiated by canonical cytokine signaling (i.e., leptin signaling in these experiments) may be linked to the initiation of hypoxic gene transcription programs in the RPD (13–15) (Figure 1B; Table 1). While greater characterization of all interactions within the transcriptome lies outside the scope of the present studies, these data suggest that leptin may function in hypoxic stress adaptation, in part, by regulating the gene expression of key glycolytic enzymes and enhancing overall glycolytic activity. Indeed, the transcriptome analyses found leptin regulates *pfkfb3*, a critical stimulator of PFK, the rate limiting enzyme for glycolysis, and the glycolytic enzyme *gapdh*. Also, this work demonstrates that the machine learning approach to transcriptomic analysis employed here may serve as a robust hypothesis-generating tool that can be used to accurately predict functional outcomes (e.g., regulation of hypoxia and glycolysis) from high throughput RNA-seq gene expression data. To our knowledge, this is the first characterization of leptin induced changes to the transcriptome and regulation of glycolytic genes in a primary cell system. However, leptin regulation of glycolytic gene transcripts has been recently described in cancer cell lines, indicating this particular function of leptin is likely conserved across vertebrate taxa and may serve an important role in tumor bioenergetics (58, 59).

A series of *in vitro* orthogonal tests confirm the findings of the transcriptome analysis and establish that leptin increases glycolysis directly at the cellular level. Several experiments conducted independently show that, while supraphysiologic doses of leptin (100 nM) applied in these studies exert profound effects on the RPD transcriptome, leptin doses within the physiological range (1–10 nM) found in tilapia and other fishes (60, 61), not only mimic the transcriptional effects seen in our RNAseq analysis, but also functionally stimulate lactate secretion, a proxy for glycolytic output, within 6 h similar to that seen with 100 nM leptin (Figures 2A, 3A). These elevations in lactate occur concomitantly with increases in *pfk1* and *gapdh* mRNA abundance (Figures 2B–D) and PFK activity (Figure 3B). PFK activity and *pfk1* mRNA expression are significantly correlated with lactate secretion (Figure 2E), consistent with PFK being a rate-limiting enzyme of glycolysis (35). The simultaneous, correlated increases in lactate, PFK activity, and *pfk1* mRNA expression suggest that leptin stimulates glycolysis in a manner dependent on increased transcription and production of PFK enzyme. Evidence indicating regulators of transcription or STAT mediate leptin actions further supports this.

Many of the actions of leptin including regulation of appetite suppression, gonadotropin secretion, and metabolism may occur through a STAT3 mediated pathway (62). The leptin receptor, which is expressed in the tilapia pituitary (46), but did not change with experimental treatment in the transcriptome analyses (Supplemental Table 5), is a cytokine receptor known to directly interact with STAT3. It is well established that, in mammalian models, binding of leptin to LepR activates JAK-2. This, in turn, promotes the phosphorylation of both JAK2 and three LepR residues which have each been linked to distinct aspects of downstream leptin signaling and biology. Phosphorylation of Tyr_1138_ is known to drive STAT3 signaling which modulates gene transcription and suppresses feeding, a known function of leptin in teleosts (34). Studies also suggest that the actions of leptin may be mediated by activation of cellular ERK1/2, as has been shown previously with stimulation of PRL release by leptin (33). Recombinant tilapia LepA significantly increased abundance of phosphorylated (activated) STAT3 in the pituitary (Figures 3D–F) and STAT inhibition with Stattic ameliorates PFK activation by leptin. It should be noted that while Stattic is a strong inhibitor of STAT3 (~85%) it may also weakly inhibit STAT5 (~35%) (48). Therefore, we cannot discount that STAT5 signaling may play a slight role relative to STAT3 in mediating leptin effects on the RPD. However, given (1) STAT5 is generally unstable under the experimental temperatures used in these studies (48), and (2) the differential inhibition of STAT3 and STAT5 by Stattic, these data largely implicate STAT3 as the primary mediator of leptin signaling in this system. This suggests that the effects of leptin on cellular glycolysis in the RPD are likely mediated, in part, through a canonical JAK-STAT leptin signaling pathway, in keeping with previous reports of elevated glycolysis in cell lines with constitutively activated STAT3 proteins (63). In parallel studies, we found that blockade with the MEK blocker (PD98059) inhibits leptin-induced ERK1/2 phosphorylation but did not alter PFK activity or lactate secretion in the tilapia RPD (data not shown).

The leptin responsiveness observed in the pituitary extends to that of another cell type, hepatocytes, in which both lactate secretion and *pfk1* mRNA are elevated in a dose dependent fashion by rtLepA treatment at 6 h *in vitro* (Figures 4A,B). However, *gapdh* mRNA expression is not changed by leptin treatment in hepatocytes. Although *gapdh* expression is not significantly correlated with glycolytic activity in either cell type examined, the increase in *gapdh* mRNA with leptin treatment in pituitary RPD may, nevertheless, be an important component of glycolytic induction in prolactin cells where a substantial amount of energy is required for cell proliferation and protein synthesis particularly during lactation in mammals and freshwater adaptation in euryhaline teleosts, where prolactin cells can comprise as much as 50% of total pituitary volume (49, 64). Indeed, our data show that leptin treatment significantly enhances the abundance of both *prl1* and *prl2* (Supplemental Figure 2) confirming responses observed *in vivo* (30). Interperitoneal and intravenous administration of leptin increases *in vivo* glucose turnover in mice, likely in part by enhancing glycolysis through an action that may involve mediation by the central nervous system (7). *In vivo* studies in tilapia show that hyperosmotic stress associated with seawater challenge elevates both leptin gene expression and circulating lactate levels suggesting that the hormone as well as other factors may be involved in enhancing glucose catabolism during stress (8). Leptin is known to suppress the hypothalamic-pituitary-adrenal axis in vertebrates (65), directly reducing ACTH secretion in teleost fish (66). Whether leptin suppression of ACTH affects glycolysis in the RPD of tilapia as a whole is unknown, however this hypothesis is amenable to further testing. Regardless, the present investigation demonstrates for the first time that leptin may directly increase cellular glycolysis, in part through increasing the production and activity of the key enzyme in glycolysis, PFK. Hence, the hormone may also work through central nervous system pathways, as well as directly on peripheral tissues as shown here, to augment glucose oxidation and overall energy expenditure in vertebrates.

A conserved function of leptin among vertebrates is promotion of energy expenditure through catabolism of energy stores and stimulation of metabolic rate (5, 10). However, the mechanisms by which leptin might accomplish these actions are only partially understood. Leptin stimulates lipolysis and beta-oxidation, providing citric acid cycle substrates that lead to increased oxidative phosphorylation and metabolic rate (4, 67). Additionally, we propose that leptin stimulation of glycolysis and subsequent pyruvate production under normoxic conditions, may drive oxidative phosphorylation, thereby providing a mechanism for increasing overall metabolic rate (68). Furthermore, increased leptin during hypoxia in humans (13), mice (15), and fish (14) might enhance glycolytic energy output essential for meeting the metabolic needs of cells in oxygen depleted environments (69). This is a particularly interesting insight, in as much as leptin is a known stimulator of proliferation in malignant cells (70, 71) that frequently show increased rates of glycolysis even in the presence of oxygen, a phenomenon known as the Warburg effect. Furthermore, cell lines with constitutive STAT3 activation show higher rates of glycolysis, indicating that the malignant cell glycolysis is mediated by STAT3 in a similar manner to leptin-induced glycolytic stimulation (18, 63, 72, 73). Therefore, it is possible that one of leptin's roles as a growth factor, may be to facilitate the Warburg effect as a means for increasing energy production in rapidly proliferating cells. Evidence also suggests that leptin may increase the activation of hypoxia inducible factor-1α (HIF-1α), a critical transcription factor for cell survival under hypoxic conditions, that is known to promote expression of glycolytic genes in breast cancer cells through both canonical and noncanonical pathways (74, 75). We show that leptin stimulates HIF-1α mRNA expression by 2.3-fold in the RPD transcriptome over that of controls (Supplemental Table 1). It is possible that leptin might work to enhance PFK activity and overall glycolysis through its regulation of HIF-1α, however, this is currently unknown and requires further study.

In summary, these results demonstrate that leptin at physiological levels stimulates glycolysis by increasing the expression of the glycolytic genes *gapdh* and/or *pfk1*, PFK activity, and overall glycolytic output through a STAT3 dependent mechanism in the pituitary RPD. While this finding does not comprehensively describe the regulation of all glycolytic genes by leptin, it does assess the crucial regulation of glycolytic function by the hormone. This finding extends to a second cell type, insofar as leptin stimulates hepatic *pfk1*, PFK activity, and overall glycolytic output, but does not enhance *gapdh* gene expression. Whether, the hormone works through a STAT3 mediated mechanism in hepatocytes to regulate glycolysis remains to be determined. Nonetheless, the stimulation of glycolysis by leptin demonstrated herein may reflect an important basal mechanism shared across vertebrates by which leptin induces energy production and expenditure under an array of physiological contexts including those linked to stressors that may include lipotoxicity, osmotic perturbation, hypoxia and tumor growth.

## Author contributions

JD and RB conceptualized and designed these studies, carried them out, and wrote the manuscript. DB, BR, AS, DL, and EG contributed through experimental design, data analyses, data interpretation, and manuscript preparation.

### Conflict of interest statement

The authors declare that the research was conducted in the absence of any commercial or financial relationships that could be construed as a potential conflict of interest.

## Supplementary material

Supplemental Figure 1 Detailed cytoscape diagrams are presented representing the relationship between genes for MMC modules.

Supplemental Figure 2 *In vitro* pituitary expression of *prl1* and *prl2* in response to leptin during 6 h incubation. Asterisks denote significance differences relative to control (mean ± SEM; ^*^*p* < 0.05).

Supplemental Table 1 Differentially expressed genes.

Supplemental Table 2 Modulated modularity input.

Supplemental Table 3 Modulated modularity output.

Supplemental Table 4 Numerical representation of the heat map from Figure 1. Each highly significant gene (1–400) is listed in the column and header row. The cross reference point of two genes indicates the Pearson correlation coefficient of the relationship between the two genes.

Supplemental Table 5 CuffDiff output.
