# Supplementary figures and images for: Leptin Stimulates Cellular Glycolysis Through a STAT3 Dependent Mechanism in Tilapia
Source: Front Endocrinol (Lausanne). 2018 Aug 21;9:465. doi: 10.3389/fendo.2018.00465 (PMC6110908; doi:10.3389/fendo.2018.00465)

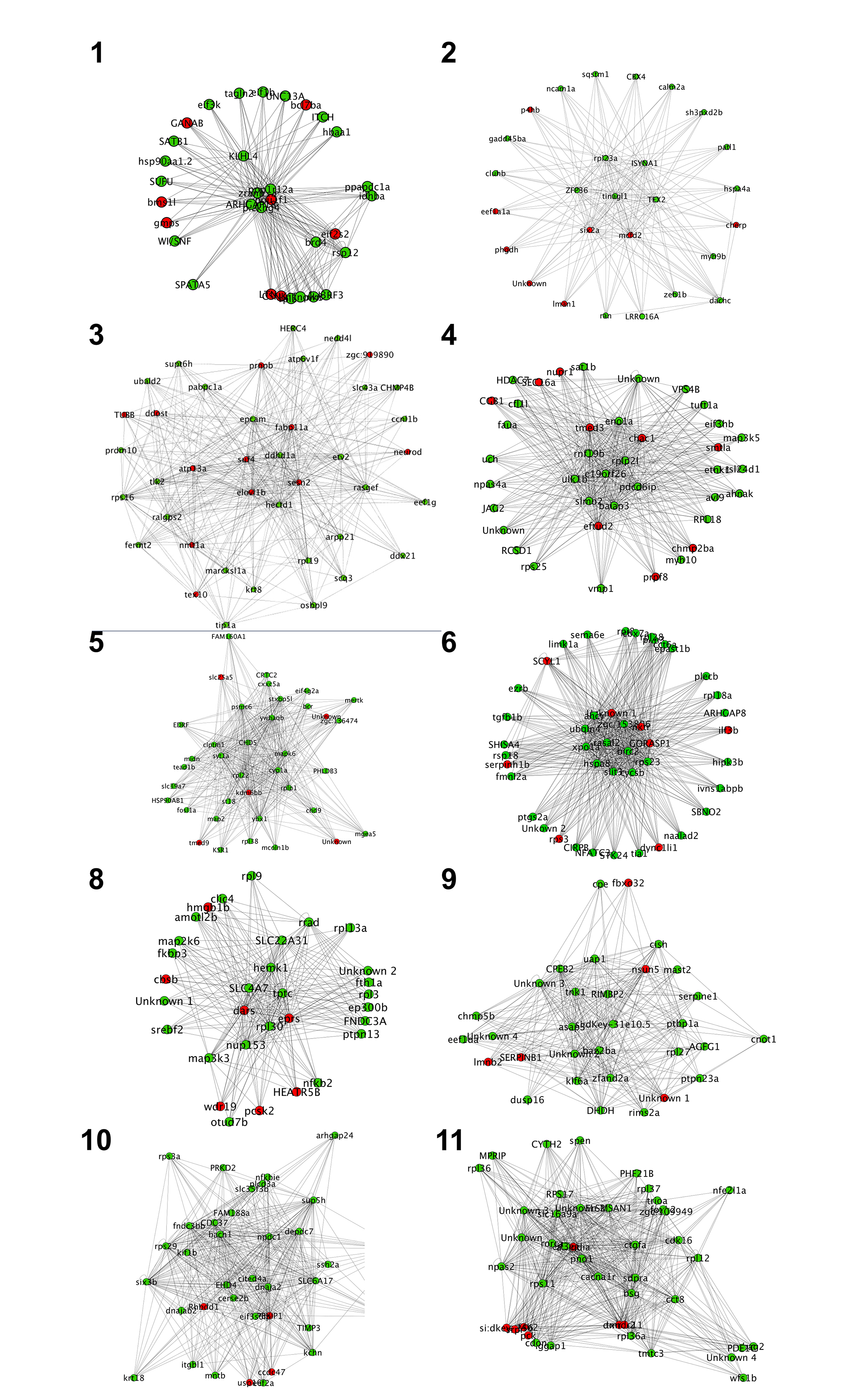

Supplement: Supplemental Figure 1 — Detailed cytoscape diagrams are presented representing the relationship between genes for MMC modules. [file Image_1.TIFF]

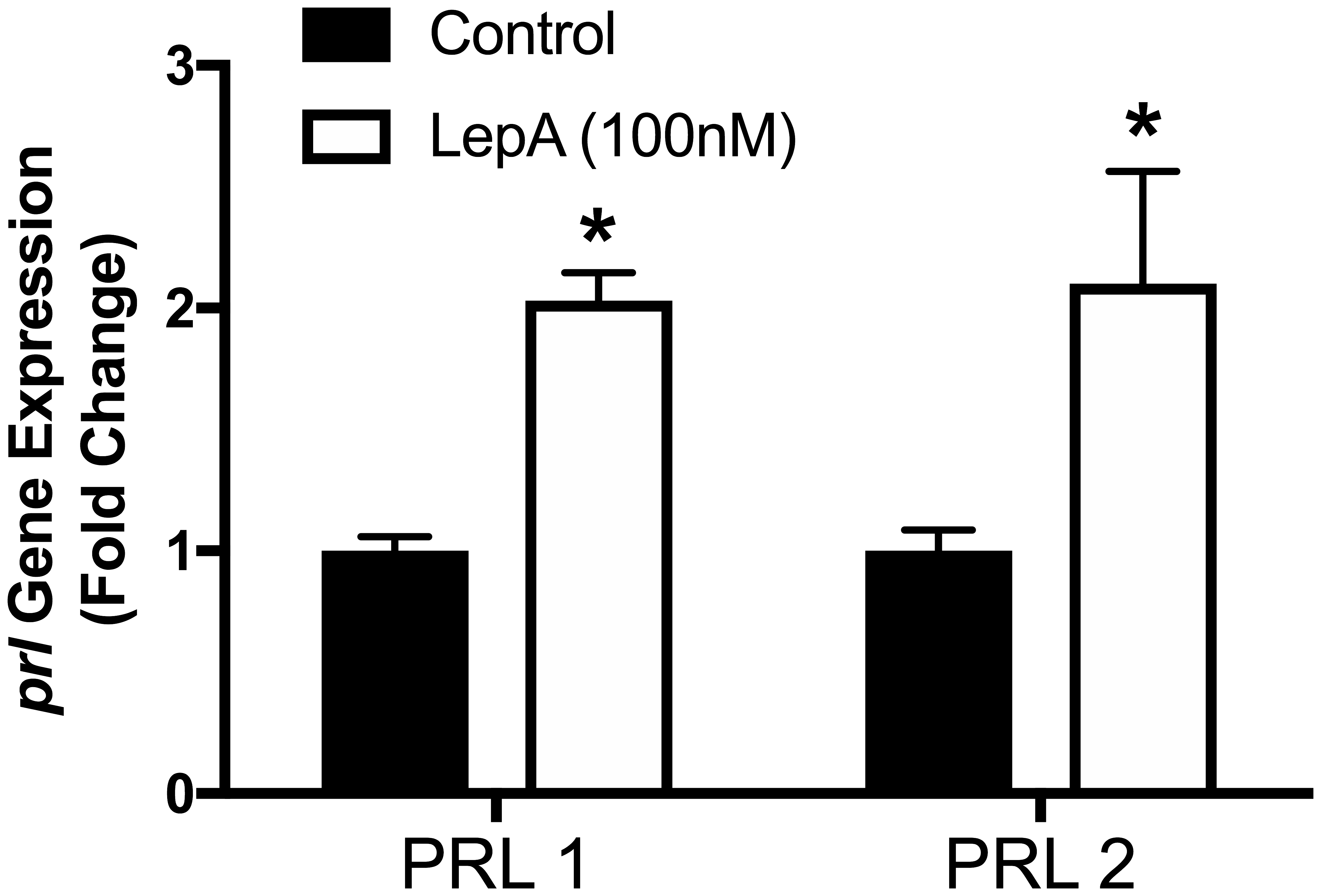

Supplement: Supplemental Figure 2 — In vitro pituitary expression of prl1 and prl2 in response to leptin during 6 h incubation. Asterisks denote significance differences relative to control (mean ± SEM; *p < 0.05). [file Image_2.TIFF]
